# Biomimetic Coating on Porous Alumina for Tissue Engineering: Characterisation by Cell Culture and Confocal Microscopy

**DOI:** 10.3390/ma8063584

**Published:** 2015-06-17

**Authors:** Elizabeth Kolos, Andrew J Ruys

**Affiliations:** Biomedical Engineering, AMME J07, University of Sydney, Sydney, NSW 2006, Australia; E-Mail: andrew.ruys@sydney.edu.au

**Keywords:** tissue engineering, porous alumina, biomimetic coating, calcium phosphate, confocal microscopy

## Abstract

In this study porous alumina samples were prepared and then coated using the biomimetic coating technique using a five times Simulated Body Fluid (5.0SBF) as the growth solution. A coating was achieved after pre-treatment with concentrated acid. From elemental analysis, the coating contained calcium and phosphorous, but also sodium and chlorine. Halite was identified by XRD, a sodium chloride phase. Sintering was done to remove the halite phase. Once halite was burnt off, the calcium phosphate crystals were not covered with halite and, therefore, the apatite phases can be clearly observed. Cell culturing showed sufficient cell attachment to the less porous alumina, Sample B, that has more calcium phosphate growth, while the porous alumina, Sample A, with minimal calcium phosphate growth attained very little cell attachment. This is likely due to the contribution that calcium phosphate plays in the attachment of bone-like cells to a bioinert ceramic such as alumina. These results were repeated on both SEM and confocal microscopy analysis. Confocal microscopy was a novel characterisation approach which gave useful information and was a visual aid.

## 1. Introduction

Tissue engineering offers a novel route for repairing damaged or diseased tissue by incorporating the patient’s own healthy cells or donated cells into temporary housings or scaffolds. Synthetic bone graft materials are seen as an off-the-shelf option that may provide a three dimensional construct for bone forming cells that behaves as a vehicle to deliver osteogenic cells and growth factors.

Porous alumina can be used as a synthetic bone graft material or a porous ceramic prosthetic device. Alumina was the first commercially significant bioceramic. It has been used in biomedical applications that require hardness, low friction and chemical stability, for example, dental implants and acetabular cup replacement in total hip prostheses.

Porosity is an important design characteristic to satisfy an application as synthetic bone graft material. Tissue ingrowth studies are used to verify the amount of porosity and the size of pores. Tissue ingrowth studies using porous alumina ceramics were first done by Hulbert in 1968 [1]. Hulbert suggests tissue ingrowth as a means of attaching prosthetic devices to the musculoskeletal system.

The incorporation of a combustible additive is the most common method to prepare porous alumina. A combustible additive can be added in the form of particles, fibres, or a continuous network of a sponge-like material. Upon sintering, the combustible additive is burnt out resulting in pores where combustible additive was present [2].

Although alumina is a biocompatible material, as it does not elicit an inflammatory response from surrounding tissue in the physiological environment, it is biocompatible by virtue of being bioinert. However, alumina is not a bioactive material as it does not have the ability to chemically bond to tissue. The body’s response to alumina is to treat it as a foreign material and encase it in a fibrous capsule.

To make the bioinert alumina material bioactive and thus suitable for the tissue scaffold application, a bioactive coating on porous alumina has been considered. One such bioactive coating is calcium phosphate. A calcium phosphate coating is considered biocompatible as it does not elicit an inflammatory or foreign body response when implanted. The application of this coating applied to the bioinert alumina may provide a bioactive layer between alumina and tissue that may lead to better anchorage.

The biomimetic coating technique produces a bioactive coating on various materials. The biomimetic coating technique is a solution soaking technique that has the ability to coat substrates of various morphologies including porous structures.

The biomimetic coating technique was first noted as a biomimetic process by Wen *et al.* in 1998 when reported a solution coating technique where a solution of calcium and phosphate ions were used to coat metal to form a calcium phosphate layer that was “similar to that of natural apatite” [3].

The biomimetic coating technique mimics the osteogenesis mechanism. This technique employs a soaking solution containing the same ions that are present in blood plasma. It is the ratio of calcium and phosphate ions in this soaking solution that enables the technique to mimic the osteogenesis mechanism. This biomimetic coating technique produces a biologically active bone-like apatite layer that is similar to the apatite formed on the surfaces of bioactive ceramics when they have been placed in contact with blood in the living body [4].

The biomimetic coating process comprises a first step of pre-treatment and a second step using a growth solution. Pre-treatment prepares a materials surface for further coating with by chemical modification (broken bonds) or physical modification (nucleation for further crystal growth). The growth solution mimics that found in the body and a simulated body fluid (SBF) is used. SBF has an ion concentration similar to that of blood plasma. See Table 2 below. The method for chemical pre-treatment was adapted from a published report [5].

In order to speed up the formation of biomimetic calcium phosphate, E Kolos utilised a five times concentrated SBF (5.0SBF) in the study on coating cotton fibres [6]. F. Barrere *et al.* found the result on titanium alloys with a 5.0SBF and CO_2_ gas was a thicker coat in a similar period and avoidance of chemical pre-treatment [7]. There are no published reports addressing cell-culturing and confocal analysis of SBF-coated alumina.

The aim of this study was to coat porous alumina samples using the biomimetic coating technique. Characterisation of this material was done using SEM, EDS, XRD, cell culture and confocal microscopy.

## 2. Method

### 2.1. Materials Used

Porous alumina, was prepared by mixing alumina with graphite. Spray-dried nanoparticulate alumina was granulated into 200 micron or 80 micron granules using a starch binder. Dry mixed with flake graphite, the flake particles were approximately 100 microns in size. Specimens were pelletised at 200 MPa into 20 mm diameter 3 mm thick pellets and sintered at 1400 °C. The result was porous alumina pellets with varying percentage of porosity. Detail for the granulation of alumina powder, mixing, pelletisation and sintering is given.

#### 2.1.1. Granulation of Alumina Powder

A total of 50 g of 0.5 µm alumina powder (calcined alumina, Alcoa Industrial Chemicals, USA code 1225) was mixed with 16 g of starch powder (Silverstar Starch, Ward, McKenzie, Australia). The dry mixture was thoroughly mixed for about 5 min with a spatula. Sufficient water was added into the mixture until it had a smooth slurry consistency and thorough stirring of the mixture continued. The slurry was then poured into a Teflon tray. The mixture and Teflon tray were placed in drying oven at 100 °C for approximately 6 h. The dried alumina cake was then crushed with a mortar and pestle. The crushed material was then run through a set of mesh sieves up to 80 µm mesh for half the samples and further sieving for the other half of the crush alumina cake up to 200 µm. Scraping with a back and forward action was used to agitate the powder through the aperture.

#### 2.1.2. Mixing of Granules

Different amounts of both 80 µm and 200 µm mesh alumina granulated powders and carbon source of graphite (Dixon No. 2, Dixon Ticondenga Company, Thomas Grozier and Son, Australia) were mixed as per Table 1. The graphite particle size was approximately 19 µm. The mixtures were shaken in a plastic container to homogenise the composition.

The mixing ratios of carbon and granulated alumina can be seen in Table 1.

**Table 1:** Mixing ratios of carbon and granulated alumina.

Mixture	%vol of C	%vol of Alumina	Density of C	Density of Alumina	Total Density	Total vol	vol of C	vol of Alumina	Mass of C	Mass of Alumina
1.00	0.00	100.00	0.00	4.00	4.00	1.00	0.00	1.00	0.00	4.00
2.00	5.00	95.00	0.09	3.80	3.89	1.03	0.05	0.98	0.00	3.72
3.00	10.00	90.00	0.17	3.60	3.77	1.06	0.11	0.95	0.02	3.44
4.00	15.00	85.00	0.26	3.40	3.66	1.09	0.16	0.93	0.04	3.16
5.00	20.00	80.00	0.34	3.20	3.54	1.13	0.23	0.90	0.08	2.89
6.00	30.00	70.00	0.51	2.80	3.31	1.21	0.36	0.85	0.18	2.37
7.00	40.00	60.00	0.68	2.40	3.08	1.30	0.52	0.78	0.35	1.87
8.00	50.00	50.00	0.85	2.00	2.85	1.40	0.70	0.70	0.60	1.40
9.00	60.00	40.00	1.02	1.60	2.62	1.53	0.92	0.61	0.93	0.98
10.00	80.00	20.00	1.36	0.80	2.16	1.85	1.48	0.37	2.01	0.30
11.00	90.00	10.00	1.53	0.40	1.93	2.07	1.87	0.21	2.85	0.08

Density of C = 1.7 g/cm^3^; Density of alumina = 4.0 g/cm; Mass of pellet = 4.0 g.

#### 2.1.3. Cold Press Pellet Production

The die was first cleaned with acetone to remove any impurities. The die was then coated with stearic acid which acts as a lubricant to ease the removal of pellets. The die was then filled with the mixture of alumina and graphite. The second ram was then put into the mould to compact the powder and placed onto the cold press machine. The pellet was then cold pressed at a pressure of 200 MPa which is about 9 tonnes on the pressure gauge. After a while the pressure was released and one ram was removed from the cold press machine. The pellet was then removed from the mould using the pellet removing machine.

#### 2.1.4. Sintering of Alumina Samples

Alumina samples were then sintered to a temperature of 1400 °C. However, first stage was sintering to 1250 °C and then further sintering at 1400 °C was done. Samples were heated in air furnaces at a heating rate of 100 °C per hour. Samples were placed in oil.

### 2.2. Pre-Treatment

A chemical pre-treatment was applied to the porous alumina. The method used in this paper can be seen below. Samples were ultrasonically washed with acetone, ethanol and distilled water. The samples were then immersed in 10 mL of concentrated sulfuric acid (*H*_2_*SO*_4_) aqueous solution in 50 mL plastic containers with plastic lids tightly secured. Plastic containers containing samples and pre-treatment solution were kept at 95 °C in a dryer for 4 days without disruption. After this treatment, samples were cooled to room temperature and washed in distilled water. Samples were then dried at 40 °C for 2 days.

### 2.3. SBF Preparation

A total of 5.0 SBF was prepared for use as a growth solution for the coating of pre-treated samples. It was prepared as from Table 2. The amounts of reagents used to attain the selected ions were calculated using molar ratios from corresponding reagents. The pH was measured and adjusted to pH of 7.4 with tris(hydroxymethyl)aminomethane and dilute hydrochloric acid (HCl).

**Table 2:** Ion concentration of 1.0 Simulated Body Fluid (SBF) and 5.0 SBF solution in comparison with those of blood plasma.

	Concentration (mM)							
	Na^+^	K^+^	Ca^2+^	Mg^2+^	HCO_3_^−^	Cl^−^	HPO_4_^2−^	SO_4_^2−^
Blood plasma	142.0	5.0	2.5	1.5	27.0	103.0	1.0	0.5
1.0SBF	142.0	5.0	2.5	1.5	4.2	148.0	1.0	0.5
5.0SBF	710.0	25.0	12.5	7.5	21.0	740.0	5.0	2.5

### 2.4. SBF Coating Trials

After chemical pre-treatment, porous alumina was treated with a SBF solution. The method employed to coat alumina in this paper is reported below. Samples of porous alumina were immersed in 30mL of acellular 5.0 SBF in plastic containers at 36.5 °C buffered to pH = 7.4. SBF was renewed every 2–3 days to maintain the ion concentration of the solution. After 16 days the samples were removed from the SBF, gently washed with distilled water, and dried at 50 °C. SBF coated porous alumina samples were heat treated to at 950 °C at a heating rate of 100 °C per hour in an air furnace.

### 2.5. Cell Culturing

#### 2.5.1. Cell Line Used

For all cell culturing in this thesis, the MG-63 human derived osteosarcoma cell line was employed for the calcium phosphate fibres and the calcium phosphate fibre tissue scaffold experiments.

#### 2.5.2. Culturing Procedures

The coated samples were seeded with human derived MG63 cells at a density of 50,000 cells per ml and cell media. The media of the cells was replaced after 4 days. After one week, the media was pipetted out of each well and the specimens were rinsed 2 times with Phosphate Buffer Solution (PBS). The specimens were then fixed with 2% glutaraldehyde rinsed with PBS, dehydrated in a series through a graded series of ethanol and critical point dried. Finally they were mounted on aluminium plates and coated with a thin layer of gold or platinum.

A twenty four day cell culture was done to observe cell integration into the porous structure.

### 2.6. Characterisation of Materials

#### 2.6.1. XRD Analysis

X-ray diffraction was carried out to determine crystallinity of coated phases and phases present. A Siemans D5000 was utilised for x-ray diffraction (XRD) with an x-ray copper tube with an accelerating voltage of 40 kV and a filament current of 30 mA. The angle of scan was 5–70°, with a step size of 0.1 and a step time of 320°.

#### 2.6.2. SEM Analysis

The sintered calcium phosphate fibres were examined in a Philips SEM 505 scanning electron microscopy (SEM) with an average accelerating voltage of 7 kV. Samples were analysed primarily on the top surface. Samples for the cell integration study were observed at the cross-section with liquid nitrogen freezing and cracking with a blade.

#### 2.6.3. EDS Analysis

The sintered calcium phosphate fibres were examined in a Philips SEM 505 scanning electron microscope (SEM) with an EDAX DX-4 EDS System (EDS) attached, operating at 7 kV.

#### 2.6.4. Porosity Measurements

Determination of porosity for porous alumina samples was done using the boiling water method as described by Australian Standards AS 1774.5 2001.

The boiling method that was followed was: test samples were dried in a drying oven at 100 °C for one hour; then cooled to ambient temperature and the dry mass $m_{D}$ was immediately recorded to nearest 0.1 g; the samples were then placed in a beaker of distilled water and boiled for 2 h then cooled overnight in immersion liquid (water); the balance was zeroed for immersion weighing with loop or saddle for immersion test; the specimen was removed from the cooled immersion liquid and was placed onto loop or saddle and lowered into the water in position beneath the balance hook (ensuring that the specimen and saddle remain immersed during weighing and that they do not touch the sides or bottom of the vessel), the mass $m_{i}$ was recorded then the sample was put into another beaker of distilled water—this step was done three times for an average; to do the saturated test the specimen was withdrawn from the beaker of distilled water and quickly drained with excess water removed with a damp cloth with care taken not to touch the sample; the specimen was then placed onto the saddle or loop and suspended in air for a saturated mass $m_{s}$ measurement, the weight was recorded after 30 s to ensure all specimens have the same conditions. The formula for apparent porosity is as follows: $P_{a}=\frac{m_{s}-m_{D}}{m_{s}-m_{i}}\times 100$.

#### 2.6.5. Confocal Microscopy

Samples from the twenty four day cell culture were analysed for cell integration using confocal microscopy. The confocal microscopy systems employed was a Leica DMIREBE inverted stand equipped with a Leica TCS2MP confocal system and Coherent Mira turnable pulsed titanium sapphire laser. The laser line excitation was 488 nm and the FITC emission spectrum was 500–588 nm for collection. The microscope used a Leica Microsystem Heildelberg GmbH computer running system. A 63.0 × 1.20 numerical aperture water correction objective lens was used and pin hole set to 0.000134 m. Samples for confocal microscopy were fixed but not fully dehydrated, samples sat in 70% alcohol. Optical sections were taken through the depth of the cell coverage with auto-florescence of cells.

## 3. Results and Discussion

Porous alumina samples utilised in this paper were made using alumina granules prepared by sieving through 200 µm or 80 µm and mixing with carbon in ratios of 5% or 60% carbon and granulated alumina. Samples were formed into pellets of roughly 20mm diameter. Samples used to observe coating efficiency with porosity is noted in Table 3.

**Table 3:** Sample Identification.

Mixing Ratio of Carbon and Granulated Alumina	Alumina Granules Prepared by Sieving Through	
	200 µm	80 µm
5%	A	
60%		B

### 3.1. Materials Characterisation

The samples were characterized with SEM, XRD and porosity measurements.

**Figure 1:**
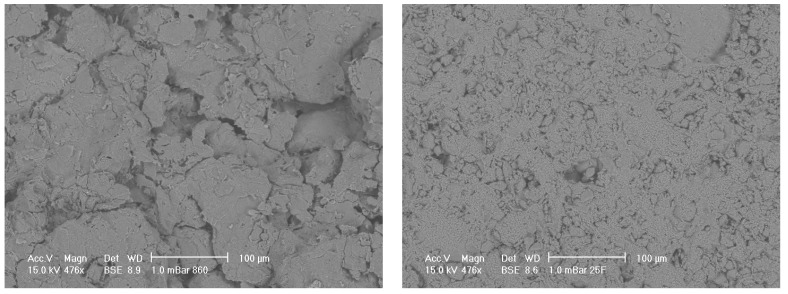
(**left**) SEM micrograph of porous alumina samples before SBF treatment for sample A with original magnification of approximately 500X. Large porous regions are evident; (**right**) SEM micrograph of porous alumina samples before SBF treatment for Sample B with original magnification of approximately 500X. Small porous regions are evident.

Figure 1 shows pellets of porous alumina after fabrication, without further treatment. Figure 1 (left) shows Sample A which is a pellet of porous alumina prepared from 80 µm mesh alumina granulated powder mixed in a ratio of 60% carbon to granulated alumina. Figure 1 (right) shows Sample B which is a pellet of less porous alumina prepared from 200 µm mesh alumina granulated powder mixed in a ratio of 5% carbon to granulated alumina with far less porosity after fabrication.

**Figure 2:**
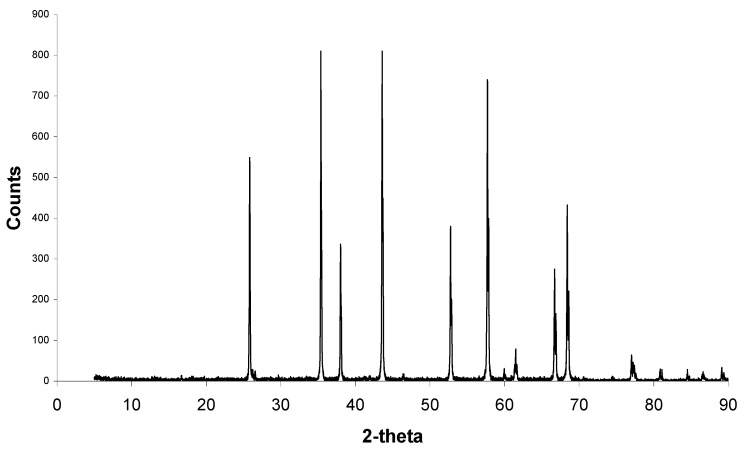
XRD pattern for alumina showing corundum peaks signalling that the alumina is a pure phase.

Figure 2 shows the XRD pattern for corundum alumina that makes up the sintered alumina pellets.

**Figure 3:**
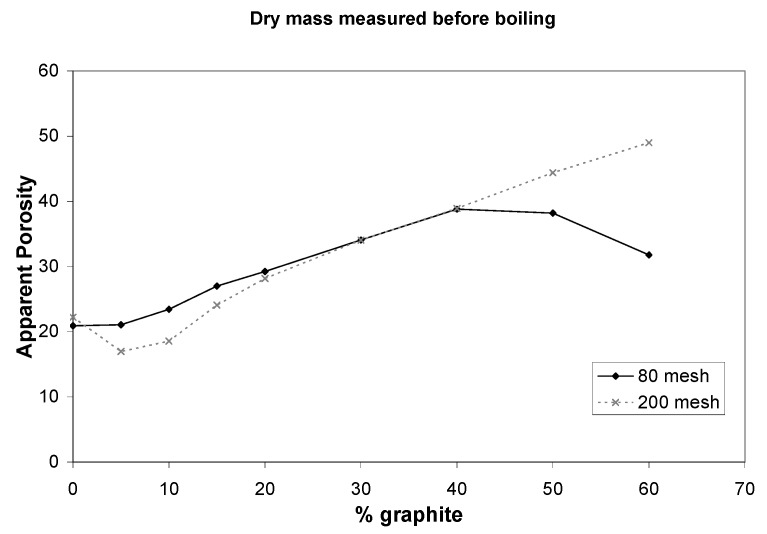

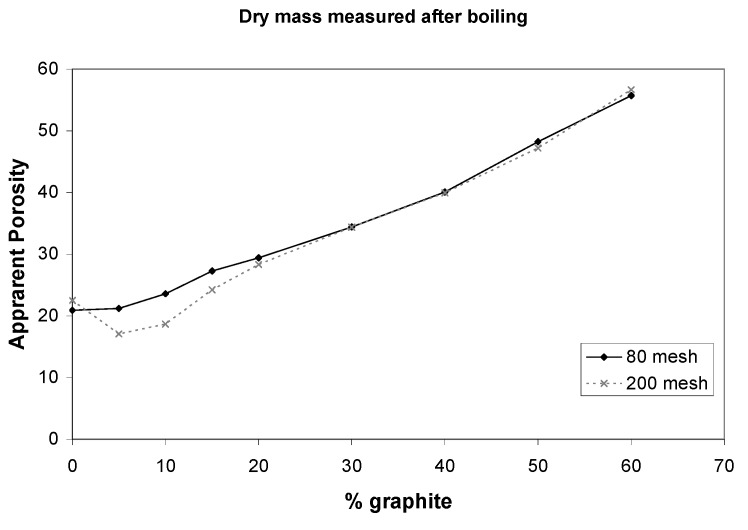
A plot presenting apparent porosity for each alumina sample for increasing % graphite component, or increasing porosity with dry mass measurements taken either before or after boiling.

Figure 3 shows two plots for porosity, dry mass measured before boiling of alumina (top) and dry mass measured after boiling of alumina (bottom). Apparent porosity increased with increasing percent graphite added to alumina mixture. Two hundred mesh samples; samples that had a smaller particle size, dipped to a lower porosity before increasing. This is mostly likely due to laminations in the pellets.

It is important to take dry mass measurements after boiling with samples of high porosity.

If dry mass measurements are taken before boiling, as suggested in the standard quoted in methods Porosity Measurements, the porosity of high porosity samples goes down. If dry mass measurements are taken after boiling, the porosity of high porosity samples increases, which is expected. This is due to fragility of porous samples especially because pieces break away during boiling. It may be surmised that 200 mesh pellets was not as sensitive to dry mass measurements being done before or after boiling.

### 3.2. Pre-Treatment

Alumina was pre-treated to follow the biomimetic technique of pre-treatment and SBF, and to instigate nucleation for calcium phosphate crystal growth.

**Figure 4:**
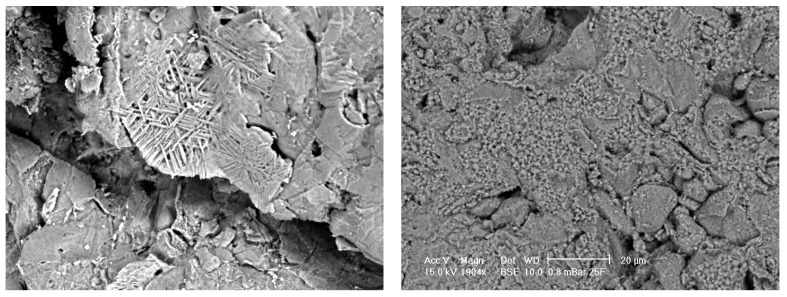
SEM micrographs of alumina control samples after pre-treatment with approximate magnification of 2000. Porous Sample A (**left**) shows acicular structure from etching. Less porous Sample B (**right**) shows fine structure from etching.

Figure 4 shows an SEM micrograph of Sample A (left) and Sample B (right) alumina pre-treated with concentrated sulphuric acid. With pre-treatment, the surface of the porous alumina was etched, that is, the grain structure is easier to identify. An acicular structure, that is, crystal structure appearing needle-like, was apparent at some porosities or % graphite and at some high original magnifications of 1904X on different samples. The surface topography was generally seen to increase with micro porosity apparent even at low % graphite, that is, low porosity samples.

The effect of pre-treatment on the alumina surface is easily seen in these SEM micrographs. It is envisaged that pre-treatment will allow more efficient SBF coating of calcium phosphate due to an increase in surface topography for heterogeneous nucleation and breaking of Al-O bonds to produce Al-OH bonds at the surface for chemical attachment of the calcium then phosphates.

The formation of Al-OH bonds is disputed. Some researchers report that Al-OH groups do not induce apatite nucleation in a body environment [8,9,10]. Other researchers report the attachment of ions to the surface is possible through segregation of bioceramics [11]. Therefore segregation, a diffusional process, may confer a degree of bioactivity on the surface of a ceramic whose bulk is inert. The calcium ions that are present on the alumina as an impurity or obtained from the SBF in a later step will thus improve bioactivity of alumina. The improvement of bioactivity of alumina will occur at the calcium ions diffuse into the surface of the alumina. Thus bioactivity may be improved due to high surface concentrations of calcium and low bulk calcium concentrations.

Researchers who have coated dense alumina using the G-glass method as pre-treatment give no indication as to the chemical attachment process for pre-treatment except that the “growth of the apatite layer is controlled by mass transport across the interface between the crystal and the fluid” [12].

A stipulation of the biomimetic technique is that pre-treatment is required for further calcium phosphate coating using a SBF solution. Alumina is a bioinert material and requires an acidic pre-treatment to prepare the surface.

Alumina was pre-treated with a concentrated acid that etched the alumina surface. Chemical integration of the OH groups was also achieved. It is probable that both physical and chemical modification occurred. Porous alumina has not been previously coated using the biomimetic coating technique. Further investigation into pre-treatments for porous alumina could be considered, especially the effect of physical modification.

### 3.3. SBF Coating

Once samples underwent pre-treatment they were soaked in SBF solutions. Materials were soaked in 5.0 SBF. Samples were characterised with SEM, EDS and XRD.

**Figure 5:**
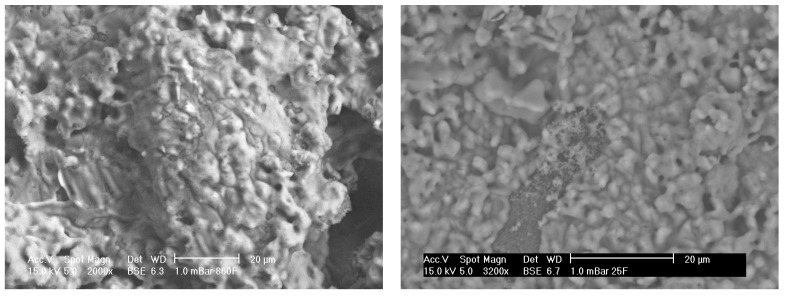
SEM micrographs of alumina after SBF soaking showing porous Sample A (**left**) and less porous Sample B (**right**) alumina. A coating is clearly visible.

Figure 5 (left) shows SEM micrographs of porous alumina Sample A that has been pre-treated and then soaked in a 5.0 SBF solution. Figure 5 (right) shows SEM micrographs of less porous alumina Sample B that has been pre-treated and then soaked in a 5.0 SBF solution.

**Figure 6:**
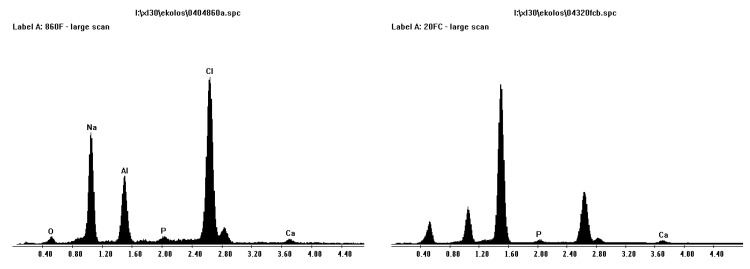
EDS plots for porous Sample A (**left**) and less porous Sample B (**right**) alumina after SBF coating. Calcium and phosphorous are present.

From the SEM analysis, there seem to be small crystals attached to the porous alumina. The coatings cover the majority of the sample, especially on the sample’s up-side. However, the crystals do not completely coat the porous surface and there seems to be areas without coating. This is possibly due to the coatings cracking off or that the coatings spread and have not yet filled the areas. On the less porous alumina Sample B in Figure 5, small needle crystals in comparison with the bigger rounder alumina particles are present. These small crystals are composed of calcium and phosphorous as suggested from elemental analysis shown in Figure 6.

Figure 6 shows EDS results from both porous and less porous alumina. Two morphologies of coating are evident on the porous alumina, one that looks amorphous that totally covers the sample (charges easily on SEM) and an acicular morphology within or under the thicker coating that is composed of calcium and phosphorous.

**Figure 7:**
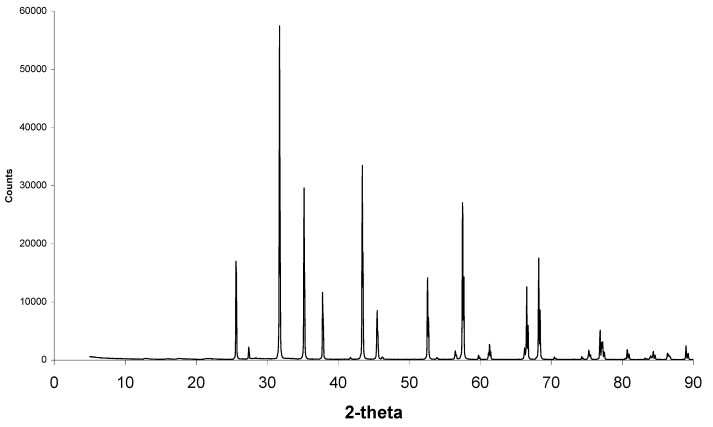
XRD scan of alumina after SBF coating showing corundum peaks and halite peaks.

Figure 7 shows an XRD scan of SBF coated alumina. The “amorphous” coating is NaCl (halite) as was suggested from XRD analysis in Figure 7. From examination of the XRD scan, the calcium and phosphorous phase is not present. Therefore, the scan was re-done with twice the step time in the hope to allow a better analysis. This result produced peaks that were slimmer, less background noise, and some phases found that matched even the smaller ones of corundum (alumina) and halite (NaCl). However there were no large peaks of calcium phosphate. This is mostly due to the small layer of calcium and phosphorous compared to alumina. This led to the opportunity to heat treat samples, burn off of the halite phase, and for sintering of the calcium phosphate phase.

Once alumina was pre-treated with sulphuric acid, the surface was etched and ready for SBF coating. The Al-OH bonds were available for apatite nuclei to form on the surface, to grow spontaneously by consuming the calcium, phosphate and hydroxide ions in the SBF. This is because the SBF is highly saturated with respect to the apatite [13]. Alumina did form the calcium phosphate layer, but the surface was not completely covered. This may be due to alumina’s poor ability to form an apatite layer following the biomimetic coating technique. Or the pre-treatment on the more porous alumina Sample A did not produce an adequate substrate for attaching, that is, the acicular structure may not be as compatible.

### 3.4. Sintering

Alumina was sintered to remove the sodium chloride phase present after soaking.

**Figure 8:**
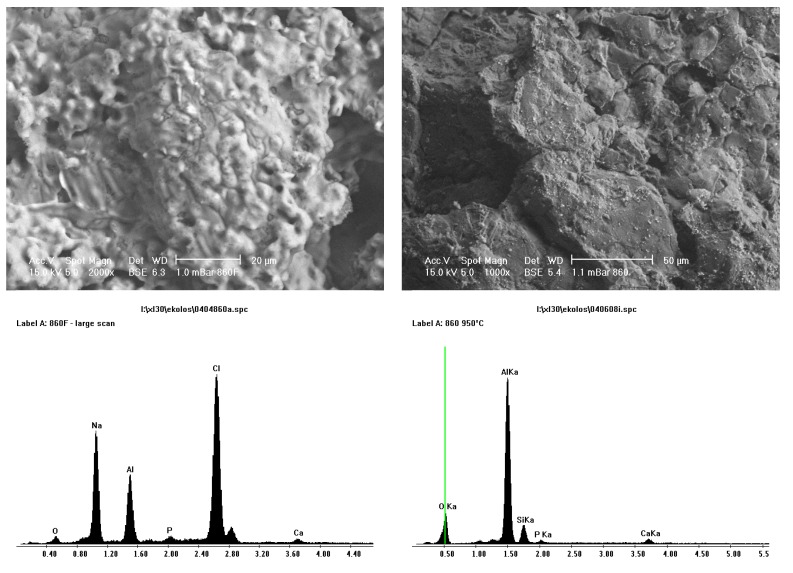
SEM and EDS of before (**left**) and after (**right**) heat treatment of coating at 950 °C for porous Sample A alumina. Sodium and chlorine are no longer present after heat treatment.

**Figure 9:**
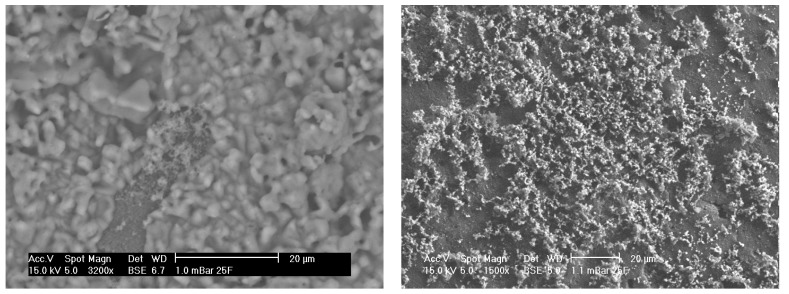

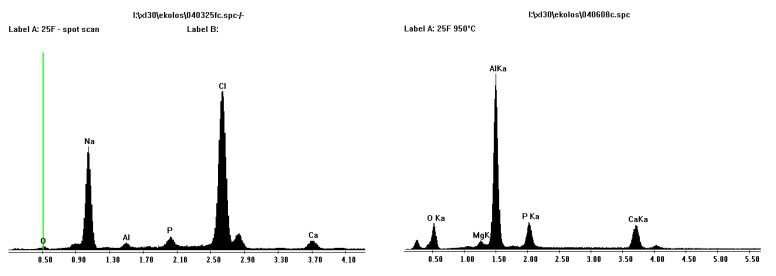
SEM and EDS of before (**left**) and after (**right**) heat treatment of coating at 950 °C for less porous Sample B alumina. Sodium and chlorine are no longer present after heat treatment.

Figure 8 and Figure 9 shows SEM and EDS images of porous Sample A and less porous Sample B alumina that have undergone pre-treatment and SBF coating on the left and sintering at 950 °C on the right. The SEM images on the left show an amorphous coating and some homogeneously dispersed small crystals that consisted of calcium and phosphorous, as seen in EDS plots. It was thought, that upon heating, the “amorphous” coating would burn out, leaving the homogeneously dispersed small crystals of calcium and phosphorous. Sintering of the alumina ceramic would not occur at this temperature, 950°C, as to sinter alumina, temperatures around 1400 °C are required. EDS results showed that this layer consisted of sodium and chlorine elements.

**Figure 10:**
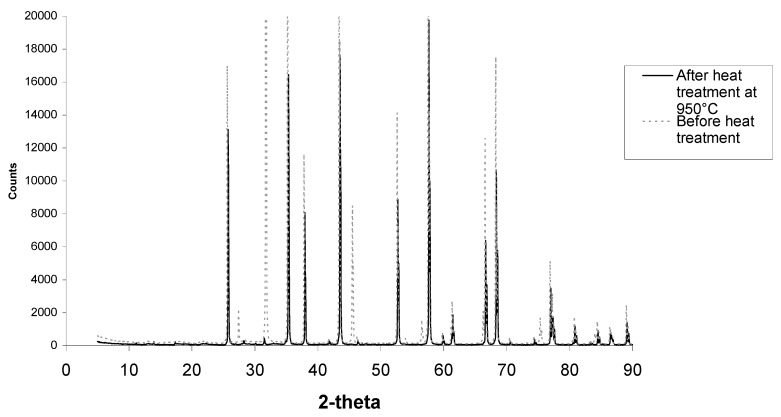
XRD scan of alumina samples before and after heat treatment showing common corundum phases and halite phases that are no longer present after heat treatment. New small peaks are present between 20 and 40°.

Further XRD analysis as seen in Figure 10 showed this phase to be halite, better known as sodium chloride. It is possible that this phase formed during SBF soaking as the 5.0 SBF has a very high percentage of sodium and chlorine ions.

On the right of Figure 8 and Figure 9, results from after sintering are presented. A sintering temperature of 950 °C was chosen, as an amorphous material would not survive this temperature, but calcium phosphate would. It is now obvious that the “amorphous” coating has gone, as seen on SEM micrographs, and no sodium and chlorine ions are present in EDS results. Small crystals of calcium phosphate are therefore apparent. The coverage of calcium phosphate crystals was more extensive on the less porous alumina Sample B then the porous alumina Sample A. The greater coverage on the less porous alumina will continue to be important in cell culture studies. Aluminium ions were present in EDS due to the calcium phosphate crystals not completely covering the alumina surface.

**Figure 11:**
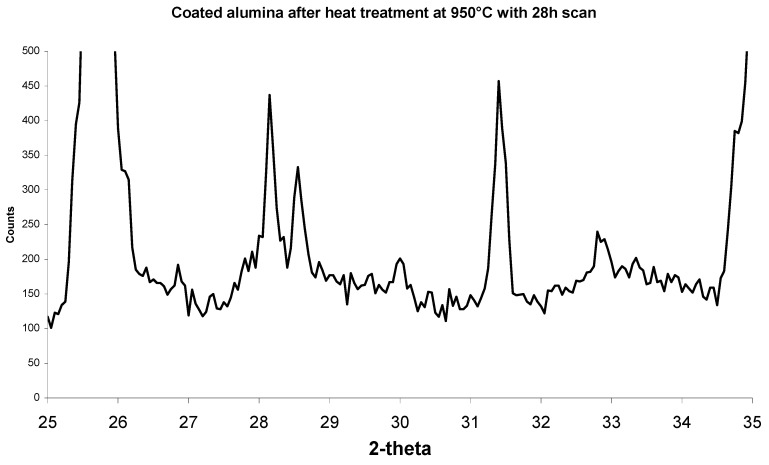
XRD scan of a select 2-theta showing a high magnification of the small peaks from Figure 10. Apatite peaks are now visible.

Figure 10 shows XRD scans of less porous alumina samples before and after heat treatment. Corundum (alumina) peaks are present at 26°, 35°, 38°, 58°, 60°, 63°, 66°, 68°, 71°, 75°, 77°, 81°, 85°, 87°, and 89°. Halite (sodium chloride) peaks are present at 28°, 32°, 45° and 56°. As seen in Figure 10 and higher magnification in Figure 11, after heat treatment there are small extra peaks at 28.1°, 28.6° and 31.4°. These phases could not be identified before heat treatment. The amounts of calcium phosphate were most likely too small and mostly covered with the halite phase to contribute to the XRD scans. Once the halite was burnt off, the calcium phosphate crystals were not covered with halite and therefore the apatite phases can be clearly observed.

Sintering was done on alumina to remove the halite layer and expose the calcium phosphate crystals. The calcium phosphate phase was exposed with sintering to 950 °C.

### 3.5. Cell Culturing

The purpose of cell culturing was to characterise the biological performance of these materials.

The porous and less porous alumina was tested with a twenty-four day cell culture. These studies were done to observe biocompatibility and cell morphology, and a further investigation was done to observe cell ingrowth.

SEM and confocal microscopy was used to view cell behaviour.

The MG-63 osteosarcoma osteoblast cell line was employed in all cell culture experiments. This was done as a standard technique for comparison purposes. Standard cell culturing procedures were followed, again for comparison purposes.

**Figure 12:**
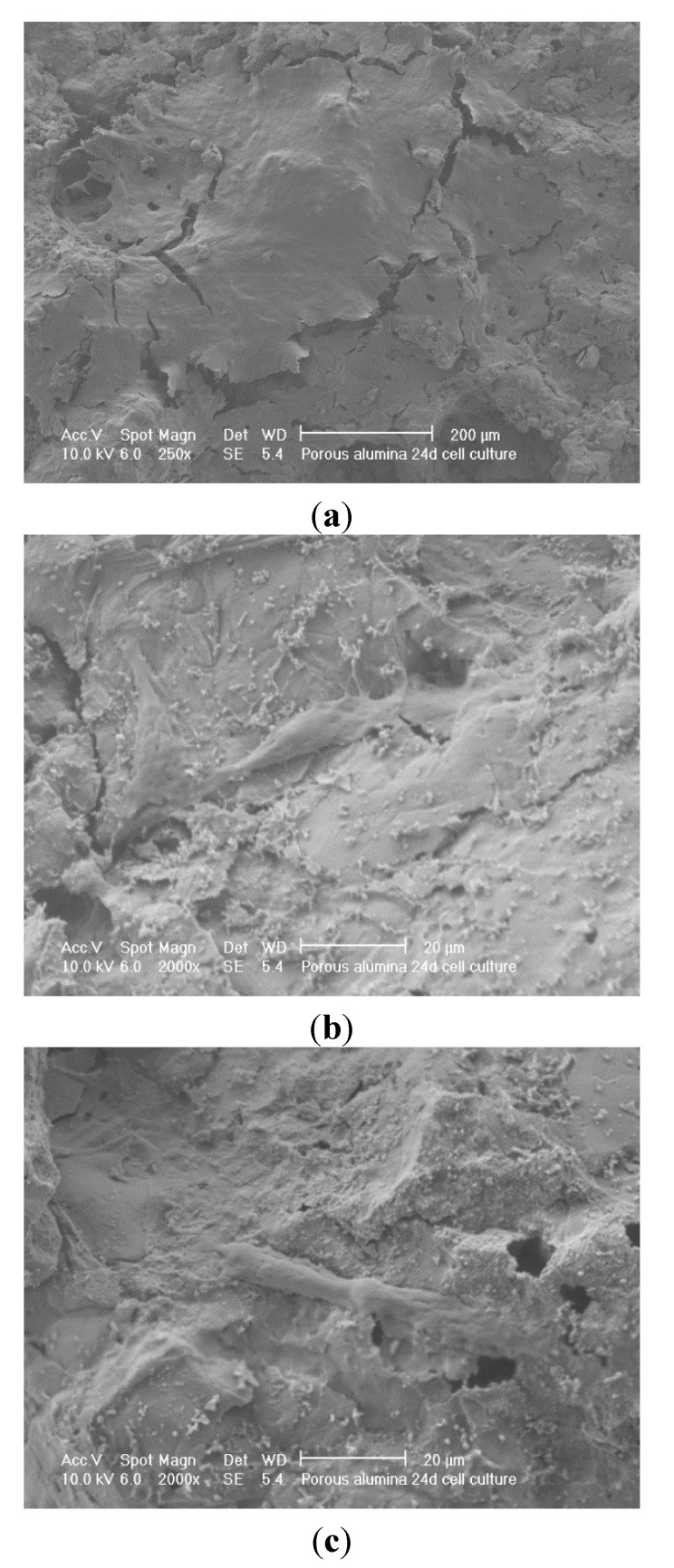
SEM micrographs of 24d culture on porous alumina Sample A; (**a**) no cells on alumina; (**b**) cells attaching to calcium phosphate particles; (**c**) single cell on alumina surface.

**Figure 13:**
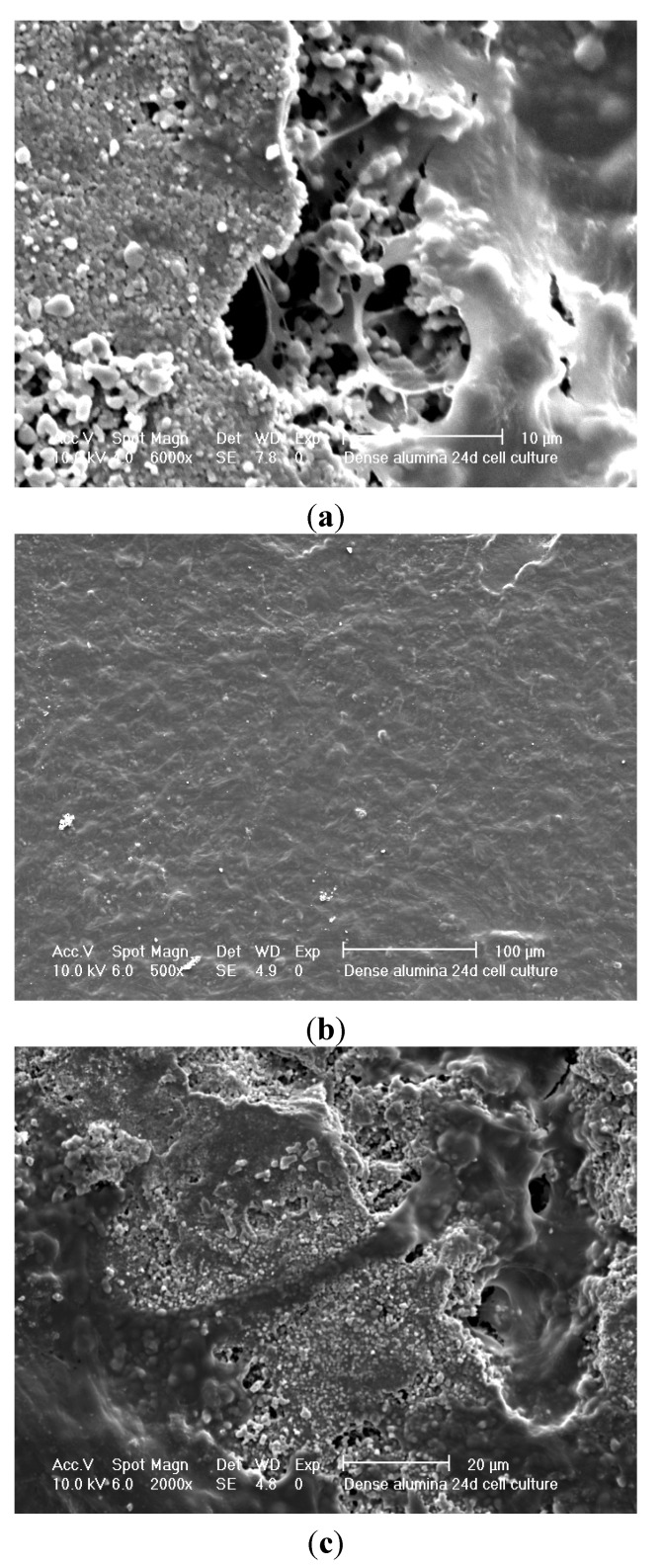
SEM micrographs of 24d culture on less porous alumina Sample B; (**a**) cells attaching in porous region; (**b**) confluent layer on ceramic surface; (**c**) cells connecting through a channel over the ceramic surface.

The porous Sample A and less porous Sample B alumina underwent cell culturing, in all cases the alumina material had been coated with a SBF coating and sintered at 950 °C. SEM and confocal microscopy were employed as characterisation tools, to examine cell attachment and cell ingrowth. Figure 12 shows SEM micrographs of a 24 day cell culture on porous alumina Sample A. Figure 13 shows SEM micrographs of a 24 day cell culture on less porous alumina Sample B. There is a confluent layer on the less porous alumina Sample B and very few cells on the porous alumina Sample A.

It is worth noting here that there was no evidence of cell penetration into the porous alumina from the confocal microscopy. The ideal outcome is a relatively thin confluent layer and significant penetration into the pores of the material. The confluent layer can be seen in Figure 13b and separate cells growths connecting in Figure 13c. Very few cells can be seen in Figure 12, only single cells that do not join in Figure 12b,c. In Figure 12b, the cell seems to be attached to the calcium phosphate particles. These calcium phosphate particles were those previously found after heat treatment of the pre-treated and SBF soaked alumina samples.

In previous findings in Figure 9 the calcium phosphate crystals are far more obvious after heat treatment especially on less porous alumina. It is probable that the osteoblast-like cells were actually attached to these calcium phosphate crystals. It was expected that the calcium phosphate crystals on the surface of the alumina would significantly aid cell attachment and proliferation.

This finding of limited but significant cellular response to calcium phosphate coated alumina, and the enhancement achieved through the calcium phosphate coating, is supported by other researchers. While no published report has addressed cell-culturing of SBF-coated alumina, published reports on calcium phosphate coated alumina coated by other methods support this general finding.

Osteoblastic cell growth has been previously successful on nano-porous alumina, with no calcium phosphate coating, with cells rapidly spreading, flattening and adhering firmly to the surface of the material [14]. Another study showed a similar result where alumina only and alumina and calcium phosphate multi-component ceramics were tested with an osteoblastic cell line. However, the initial cell-attachment efficiency was better with the calcium phosphate coating in a five day cell culture, and the differentiation of osteoblastic cells was better with the alumina samples with calcium phosphate coatings [2,15].

Confocal microscopy was chosen as a technique to view the cell ingrowth through the ceramic sample. The osteoblast-like cells were auto-florescent and therefore a wavelength could be chosen to view the cells only and not the ceramic. Therefore the results display the thickness of the cellular layer and any cell ingrowth into the ceramic and away from the confluent layer will be apparent.

**Figure 14:**
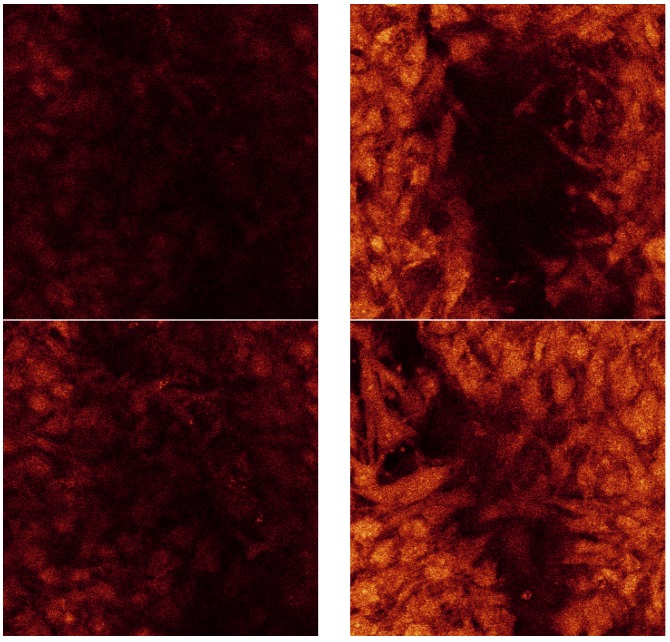

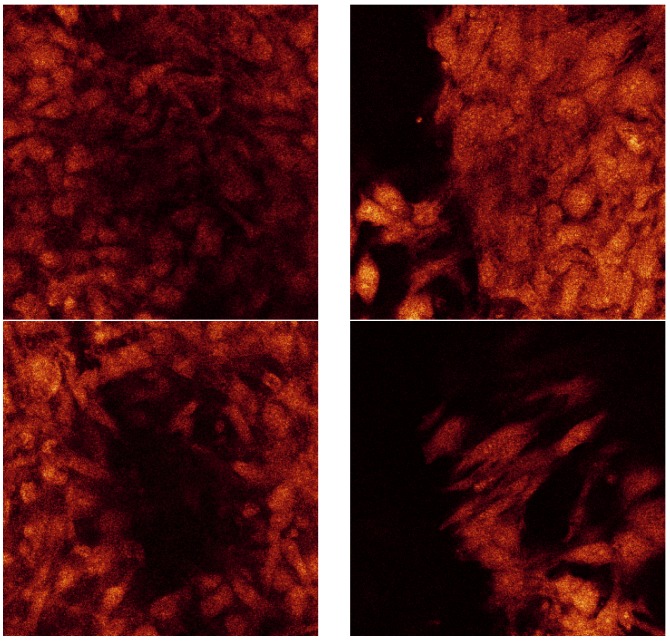
Confocal raw data series taken at random distance through the cell layer on the SBF coating on less porous alumina Sample B.

Figure 14 is a confocal raw data series displayed from consistent displacements through the thickness of the less porous alumina Sample B. The dimensions of each of these sections are 238 µm by 238 µm. The section thicknesses are ~0.4 µm as there were 173 Sections through a depth of 69 µm.

**Figure 15:**
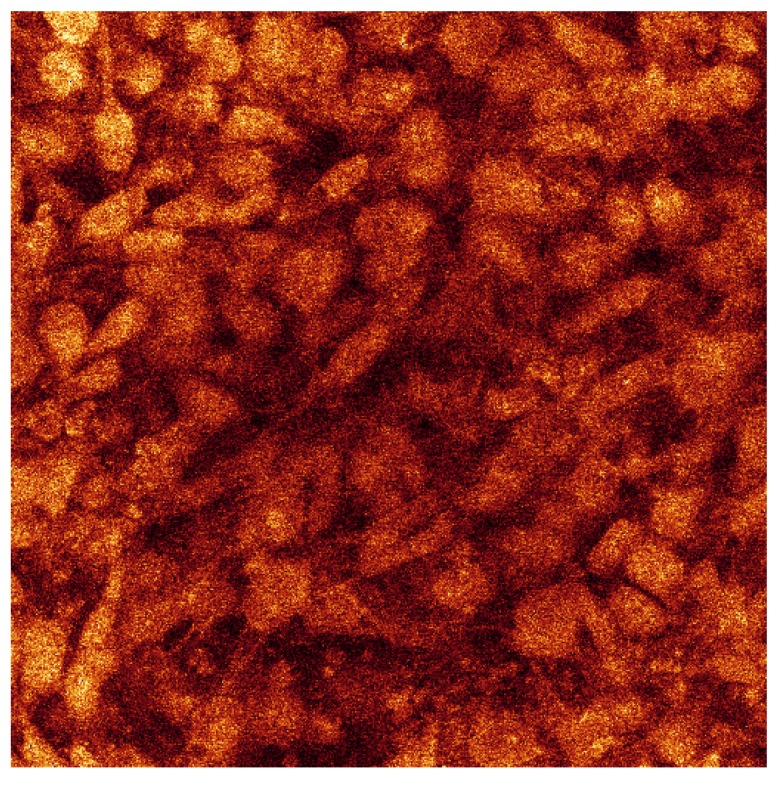
Confocal microscopy image all 173 sections for cell culture on SBF coating on less porous alumina Sample B.

Figure 15 shows the agglomeration of the separate raw data images all in one picture.

**Figure 16:**
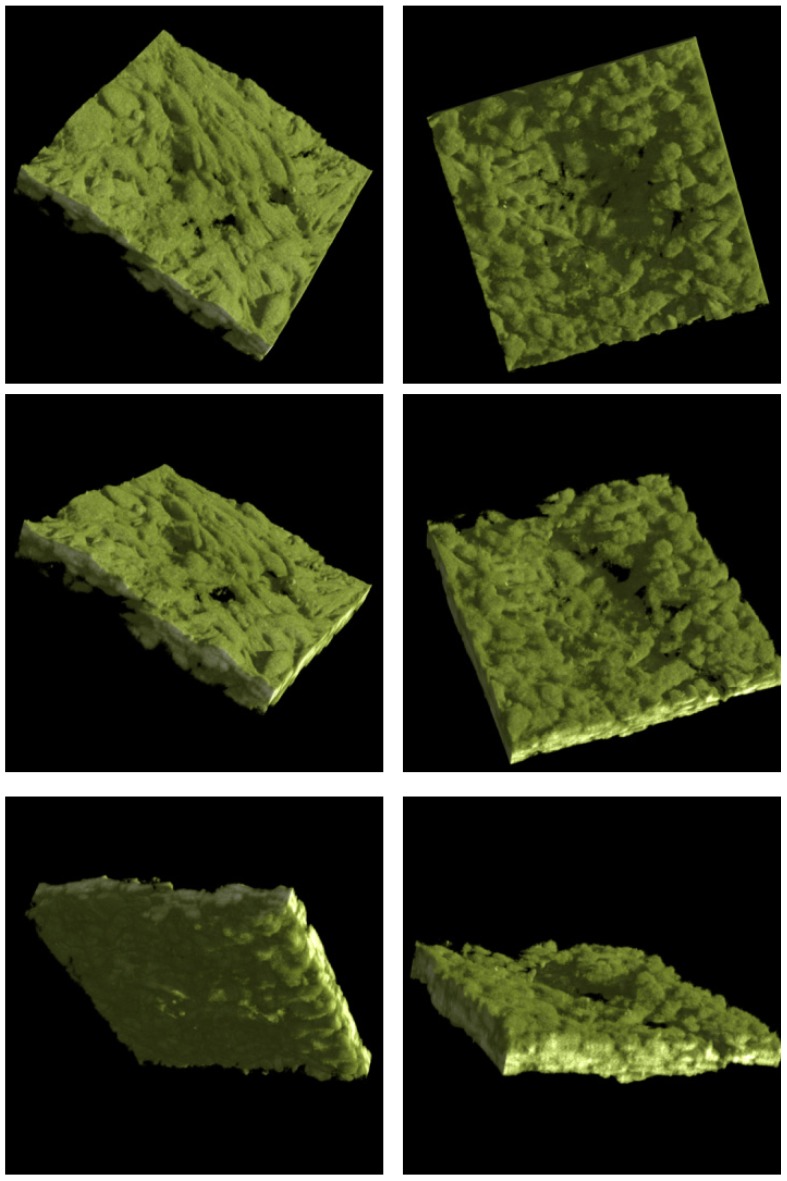
3D rendered confocal image of less porous alumina Sample B with SBF coating that was sintered with 24d cell culture.

Figure 16 is the 3D rendered images from the raw data series.

**Figure 17:**
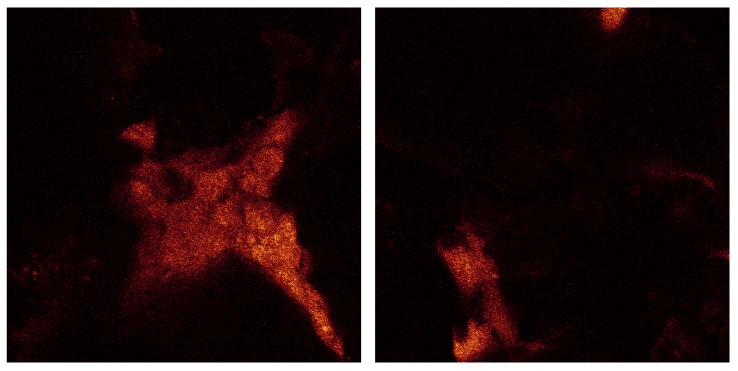
Confocal microscopy image of cells on SBF coating on porous alumina Sample A. There is very little cell coverage even after 24 day cell culture. Results confirm SEM analysis.

Figure 17 shows the confocal image taken of analysis of the porous alumina Sample A. The image on left has a length and width of 238 µm and cross-section depth of 69 µm. The image on right has a length and width of 238 µm and cross-section depth is 102 µm.

The dimension of each of these sections is 238 µm by 238 µm. For the series displayed in Figure 14 the section thicknesses are ~0.4 µm as there were 173 sections through a depth of 68 µm. This indicates that the cell layer thickness on the less porous alumina was approximately 68 µm. The alumina sample is not included in this calculation as the confocal image does not scan the ceramic. The cell layer on this sample appears to be thick, especially as viewed on the 3D rendered series in Figure 16. This series shows the cell morphology as it contours to the surface of the ceramic. It appears that the attached cells are in multiple cell layers. This gives an indication of cell thickness, if a bone cell is approximately 10 µm in dimension and the cell layer is approximately 60 µm, then the cell layer may be up to six cells deep.

The 3D rendered images in Figure 16 also present information of cell ingrowth. There appears to be only a little cellular ingrowth away from the confluent layer. This is expected as it was a less porous alumina sample that was tested. Large porous regions were not available for cellular ingrowth. Possibly the cells followed the micro-porosity of the surface and therefore matched the surface topography. The cells have covered the alumina sample including a bump in the centre of the images.

In the rendered series, this topography is evident on the cellular growth. The top cell layer is evident by the typical cellular shapes of elongated cells. The underside of the cell layer, the side closest to the ceramic, has a different cellular morphology. The cells that are closest to the ceramic have a more round and less elongated morphology. The spinning series shows this phenomenon well. This is probably contributed to by the alumina surface. This is expected behaviour in a multi-layer.

Confocal analysis was also done on the porous alumina Sample A with less calcium phosphate crystals present after heat treatment. The results show very little cell coverage after the 24 day cell culture. During culture, the cell media in the sample wells did not turn the same pink-orange as the other samples. The alumina component of the sample may have had a negative effect, especially with less calcium phosphate present unlike the less porous alumina samples. Therefore, no further confocal analysis was done on these samples as the cell coverage was poor compared to the less porous alumina sample.

## 4. Conclusions

Pre-treatment and SBF were utilised via the biomimetic coating technique to deposit a calcium phosphate coating on alumina. A coating was achieved after pre-treatment with concentrated acid. However a better coating was attained for the less porous sample Sample B. A possible reason for this may be the needle-like crystal structure formed by pre-treatment on the porous alumina Sample A may not be compatible with SBF coating. Re-investigation of the pre-treatment of the samples would be beneficial as higher porosity materials would be more useful for tissue engineering applications.

From elemental analysis, the coating contained calcium and phosphorous, but also sodium and chlorine. Halite was identified by XRD, a sodium chloride phase. Sintering was done to remove the halite phase. Once halite was burnt off, the calcium phosphate crystals were not covered with halite and therefore the apatite phases can be clearly observed.

The results show sufficient cell attachment to the less porous alumina Sample B that has more calcium phosphate growth, while the porous alumina Sample A with minimal calcium phosphate growth attained very little cell attachment. This is likely due to the contribution that calcium phosphate plays in the attachment of bone-like cells to a bioinert ceramic such as alumina. These results were repeated on both SEM and confocal microscopy analysis.

Confocal microscopy gave useful information and was a visual aid.

The overall result was that there was limited cell ingrowth with the limitation due to interconnectedness of pores and the testing medium of cell culture.
